# Elucidating the NB-UVB mechanism by comparing transcriptome alteration on the edge and center of psoriatic plaques

**DOI:** 10.1038/s41598-023-31610-y

**Published:** 2023-03-16

**Authors:** Suphagan Boonpethkaew, Jitlada Meephansan, Sasin Charoensuksira, Onjira Jumlongpim, Pattarin Tangtanatakul, Jongkonnee Wongpiyabovorn, Mayumi Komine, Akimichi Morita

**Affiliations:** 1 Division of Dermatology, Chulabhorn International College of Medicine, Thammasat University, Rangsit Campus, Klong Luang, Pathum Thani 12120 Thailand; 2 Department of Transfusion Medicine and Clinical Microbiology, Faculty of Allied Health Sciences, Chulalongkorn University, Bangkok, 10330 Thailand; 3 Department of Microbiology, Faculty of Medicine, Center of Excellence in Immunology and Immune-Mediated Disease, Chulalongkorn University, Bangkok, 10330 Thailand; 4 Department of Dermatology, Jichi Medical University, 3311-1 Yakushiji, Shimotsuke, Tochigi 329-0498 Japan; 5 Department of Geriatric and Environmental Dermatology, Nagoya City University Graduate School of Medical Sciences, Nagoya, 467-8601 Japan

**Keywords:** Molecular biology, Molecular medicine

## Abstract

Narrow band-ultraviolet B (NB-UVB) is an effective treatment for psoriasis. We aim to generate a potential mechanism of NB-UVB through comparing the transcriptomic profile before and after NB-UVB treatment between the peripheral edge of lesional skin (PE skin) and the center of lesional skin (CE skin) on the basis of molecular mechanisms of these two areas display different downstream functions. More than one-fourth of the NB-UVB-altered genes were found to be plaque-specific. Some of them were psoriasis signature genes that were downregulated by NB-UVB in, both, PE and CE skin (core alteration), such as *IL36G*, *DEFB4A/B*, *S100A15*, *KRT16*, and *KRT6A.* After NB-UVB treatment, the activity score of upstream cytokines, such as interferons, interleukin (IL)-6, IL-17, and IL-22 in pathogenesis decreased. In addition, NB-UVB could restore normal keratinization by upregulating *LORICRIN* and *KRT2*, particularly in the CE skin. Finally, we illustrated that NB-UVB is capable of suppressing molecules from the initiation to maintenance phase of plaque formation, thereby normalizing psoriatic plaques. This finding supports the usefulness of NB-UVB treatment in clinical practice and may help in the development of new treatment approaches in which NB-UVB treatment is included for patients with psoriasis or other inflammatory skin diseases.

## Introduction

Narrow-band ultraviolet-B (NB-UVB) is the first-line phototherapy for moderate to severe psoriasis (> 10% of the body surface area). Among phototherapy modalities, NB-UVB efficacy is lower than that of oral psoralen plus UV-A (PUVA). However, it has higher patient tolerability and lower adverse events than those of PUVA^1^. NB-UVB treatment results in rapid lesion clearance, long remission intervals, and minimal acute adverse events^2–4^. Although novel biologics have a high rate of psoriasis area severity index (PASI)-75 accomplishment, NB-UVB treatment may be safer than these in conditions, such as pregnancy, children, and breast-feeding mothers, based on their long-term record^1,5^. NB-UVB treatment requires no laboratory monitoring and is more affordable than biologics^1^. Moreover, it can be administered in combination with topical steroids, topical vitamin D derivative, methotrexate, acitretin, or even biologics to achieve a high PASI reduction^6–9^. These factors still make NB-UVB a useful modality in clinical practice.

The NB-UVB mechanism is complex. Apoptosis of epidermal T cells appears to be one of the cytotoxic effects of UVB radiation in inflammatory dermatoses treatment. The key cytotoxic effect of UVB during plaque clearance in psoriasis is apoptosis of epidermal keratinocytes^10^. Furthermore, UVB damages nuclear DNA (a UVB’s chromophore), which suppresses DNA synthesis in psoriatic epidermal cells^11^. In addition to its suppressive effect on keratinocytes, NB-UVB reduces the number of inflammatory myeloid dendritic cells (mDCs) and interleukin (IL)-17/22-producing CD3+ T cells^12^. In contrast, it increases the number of peripheral blood T regulatory (T reg) cells with restoration of their function in patients with psoriasis^3^. NB-UVB suppresses type I and type II interferons (IFNs), and T helper (Th) 17 signaling pathways^13^. NB-UVB treatment suppresses serum levels of psoriasis-driving cytokines, such as tumor necrosis factor (TNF)-α, IL-8, IL-12, IL-17, IL-22, IL-23, and IL-34 and elevates the levels of IL-10, an anti-inflammatory cytokine, in patients with psoriasis^14,15^. Moreover, NB-UVB treatment significantly suppresses the mRNA levels of nuclear factor-kappa-B inhibitor zeta, serpin family B member 4 (*SERPINB4*), autophagy related 13, and cathepsin S in the edges of psoriatic plaques^16^ and may attenuate TLR-3/4/9 activity in psoriatic plaques^17^.

Previously, it has been proposed that the edges of plaques show more intense inflammation than that at the center, where plaque formation is initiated; however, the center of the plaques are more stable and bioinformatic analysis reveals higher activity of growth factors at the center than that at the edges^18,19^. These results indicate that the molecular mechanisms in the different areas of plaques vary. Hence, this study aimed to elucidate the new approach comparing the molecular changes after NB-UVB treatment inside the plaque's edge with those at the center using RNA-seq and a new bioinformatic analysis program. Our results elucidate the overall concept of NB-UVB mechanism along the chronological mechanism of plaque formation, explaining how NB-UVB is effective in clinical practice.

## Results

### RNA-seq visualization

#### Immune cell alteration is indicated inside the plaques after NB-UVB treatment

**Figure 1:**
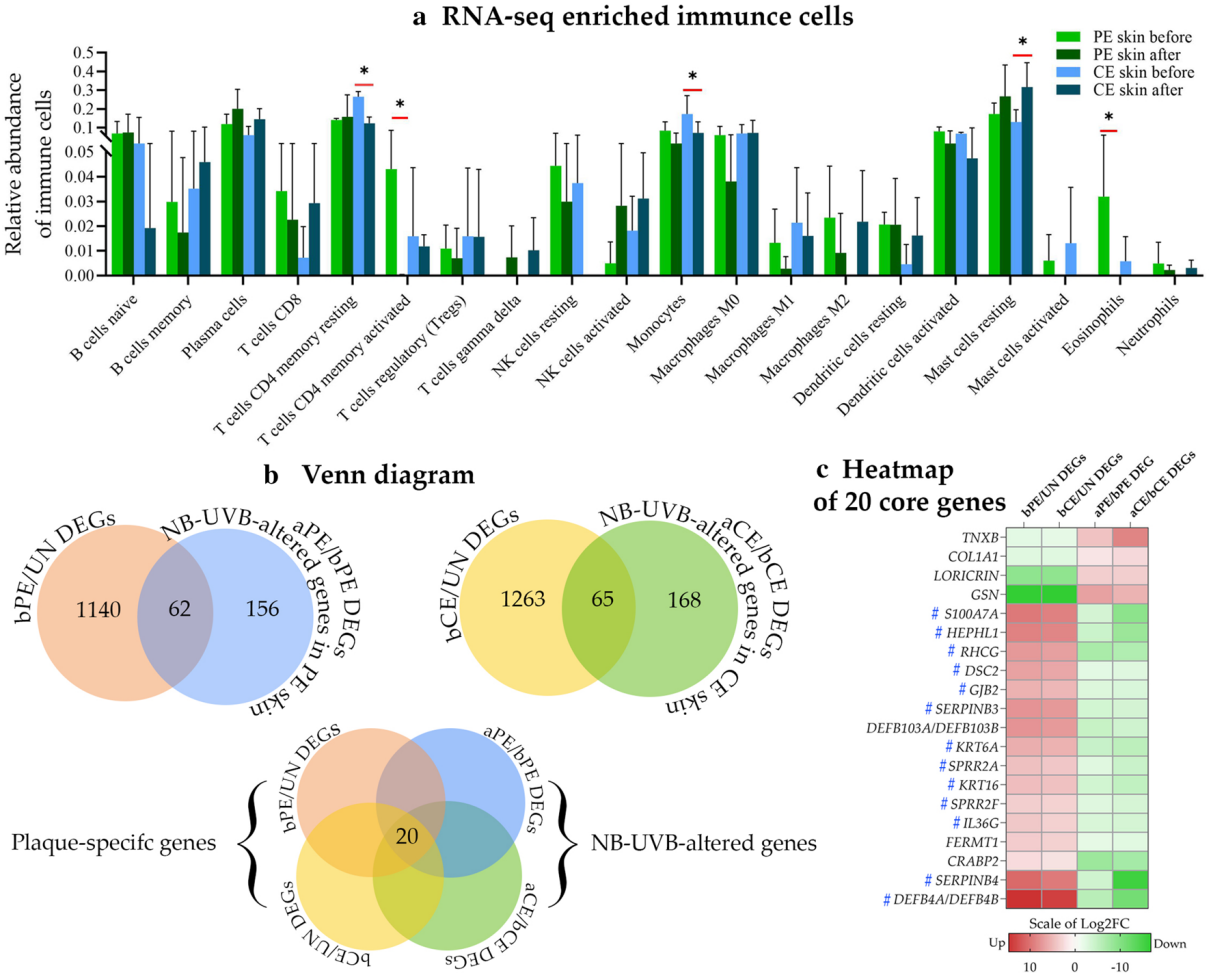
RNA-seq visualization. (**a**) Relative abundance of immune cells comparing before and after NB-UVB treatment in the PE skin and CE skin. Immune cell deconvolution was performed using the LM22 gene signature matrix and CIBERSORTx algorithm. The graph shows the mean and standard deviation of relative abundance. Significance was analyzed using the LIMMA package in Bioconductor. *p < 0.05. (**b**) Venn diagram showing the intersection implicating the number of altered plaque-specific genes. **(c)** A heatmap showing 20 NB-UVB-altered core genes. **#**, indicates the psoriasis signature genes. *aCE skin* After-treatment CE skin, *aPE skin* After-treatment PE skin, *bCE skin* Before-treatment CE skin, *bPE skin* Before-treatment PE skin, *DEGs* Differentially expressed genes, *FC* Fold change, *NB-UVB* Narrow band ultra violet B, *UN skin* Uninvolved skin.

Transcriptome data were used to analyze the relative immune cell alterations after NB-UVB treatment inside the peripheral edges of lesional (PE) skin [before NB-UVB-treated PE (bPE) skin vs after NB-UVB-treated PE (aPE) skin] and the center of lesional skin CE skin [bCE skin vs aCE skin]. The results are shown in Fig. 1a. Activated CD4+ T cells and M1 macrophage were likely to show increased suppression in the PE skin compared to that in the CE skin. Activated natural killer (NK) cells were likely to be restored in, both, the PE and CE skin. Activated dendritic cells (DCs) tended to be more suppressed in the CE skin than that in the PE skin. However, except for activated CD4+ T cells, these findings were not statistically significant.

#### NB-UVB could reverse several psoriasis signature genes as a core alteration of the edge and center of plaques

Transcriptome data were analyzed into differentially expressed genes (DEGs) using a cut-off of *p*-value < 0.01 and │log2 fold change (FC)│ ≥ 1.5. Gene expression in psoriatic plaques was classified into the following two groups: untreated group (bPE/uninvolved (UN)—and bCE/UN skin-derived DEGs) and NB-UVB-treated groups (aPE/bPE—and aCE/bCE skin-derived DEGs). DEGs of both the groups were intersected to identify the alteration of plaque-specific genes. A Venn diagram shows intersection of both the groups (Fig. 1b). The intersection indicated that NB-UVB could not alter all DEGs in untreated plaques. Only 62 (28.44%) and 65 (27.89%) NB-UVB-altered DEGs were plaque-specific for the PE and CE skin, respectively. These NB-UVB-altered DEGs are listed in Supplementary Fig. S1. Among them, 20 DEGs were identical between the PE and CE skin, representing the core alteration DEGs (Fig. 1c). Based on transcriptomic analysis lituratures^20,21^, majority of the core alteration DEGs were psoriasis signature genes (genes that tend to be more specifically expressed in psoriasis than other skin diseases or play a well-known role in psoriasis) (e.g., *IL36G*, *DEFB4A/B*, *S100A7A (S100A15)*, *SERPINB3*, *SERPINB4*, *KRT16*, *KRT6A, DSC2,* and *GJB2*).

### DEG analysis

#### NB-UVB suppressed the inflammatory processes and restored the normal differentiation and proliferation of keratinocytes in psoriatic plaques

**Figure 2:**
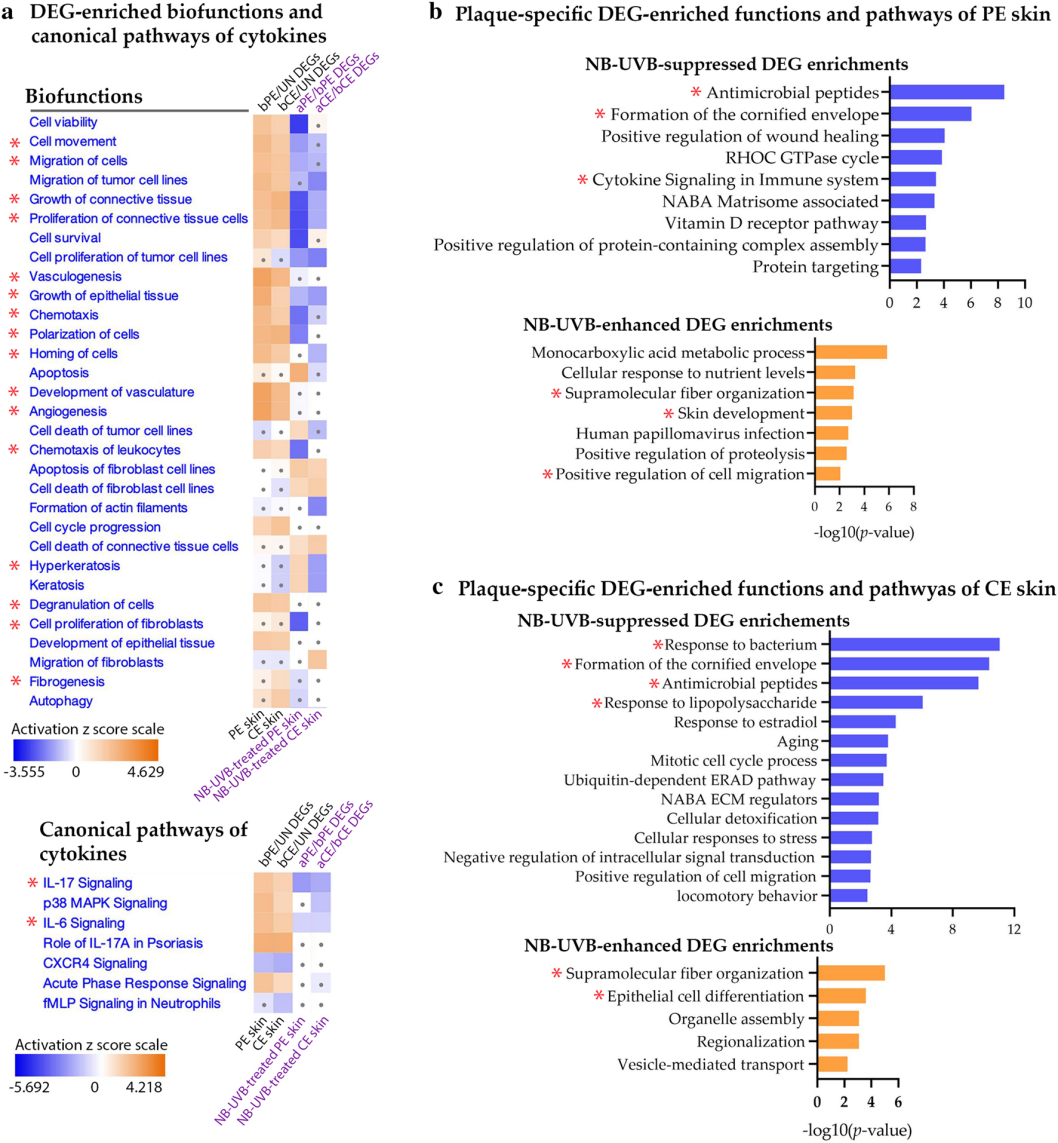
Downstream function analysis. (**a**) DEGs were analyzed for their downstream function and canonical pathway enrichment using QIAGEN’s IPA. The comparison among each set of DEGs is shown with a heatmap of activation z score. Orange, white, and blue colors represent activation, neutral, and inhibition, respectively. A dot is designated for │z score│ < 1. (**b**–**c**) Function and pathway enrichment of NB-UVB-altered plaque-specific DEGs of PE and CE skin were analyzed using Metascape. Interesting functions are designated with *. *aCE skin* After NB-UVB-treated PE skin, *aPE skin* After NB-UVB-treated CE skin, *bCE skin* Before NB-UVB-treated center of lesional skin, *DEG* Differentially expressed gene, *bPE skin* Before NB-UVB-treated peripheral edge of lesional skin, *UN skin* Uninvolved skin.

Further, we analyzed DEG-enriched biofunctions and canonical pathways of cytokines using QIAGEN’s Ingenuity Pathway Analysis (IPA) (Fig. 2a and supplementary Fig. S2). The activation z-score of functions and cytokine signaling pathways associated with psoriatic plaque development, such as chemotaxis, angiogenesis, vasculogenesis, epithelial tissue proliferation, connective tissue proliferation and growth, IL-17 signaling, and IL-6 signaling, inversely decreased with NB-UVB treatment in, both, PE and CE skin. In addition, we used 62 and 65 NB-UVB-altered plaque-specific DEGs of both PE and CE skin, respectively (supplementary Fig. S1) to analyze functions and pathway enrichments using Metascape (Fig. 2b–c). NB-UVB treatment suppressed plaque-specific DEGs of, both, the PE and CE skin which were enriched in function related to formation of the late cornified envelope (e.g., *KRT6A*, *KRT16*, which are keratin induced in inflammatory conditions) suggesting the suppression of inflammatory phenotype of keratinocytes and abnormal keratinization process in, both, the PE and CE skin. In addition, *LORICRIN* of, both, the PE and CE skin and *KRT2* of CE skin, which are related to skin development and epithelial differentiation functions, were restored, reflecting the normalization of keratinocyte differentiation. NB-UVB-altered plaque-specific DEGs in each function and pathway are listed in supplementary Table S1.

#### Core plaque-specific DEGs may be reversed either in PE or CE skin, with improvement in plaque phenotype

**Figure 3:**
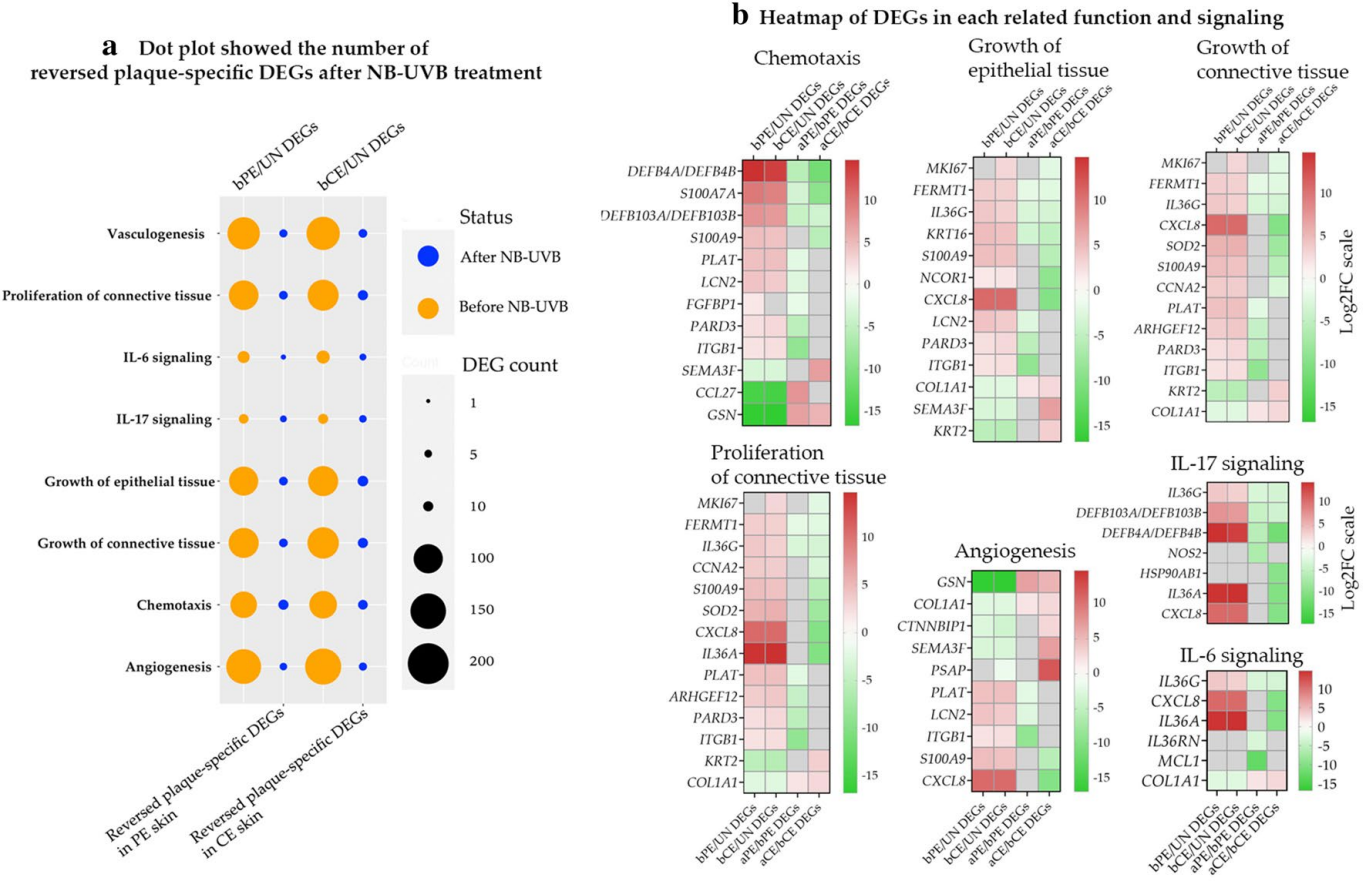
Reversed plaque-specific DEGs after NB-UVB treatment. (**a**) Dot Plot showing the number of reversed plaque-specific DEGs after NB-UVB treatment. (**b**) Heatmap of Log_2_FC showing examples of genes in each related function and signaling. Red color represents upregulated DEGs and green represents downregulated DEGs. *aCE skin* After-treatment CE skin, *aPE skin* After-treatment PE skin, *bCE skin* Before-treatment CE skin, *bPE skin* Before-treatment PE skin, *DEGs* Differentially expressed genes, *FC* Fold change, *NB-UVB* Narrow band ultra violet B, *UN skin* Uninvolved skin.

We selected some psoriasis-related downstream functions and signaling from downstream function analysis (Fig. 2) to identify the number of plaque-specific DEGs in each function that were reversed by NB-UVB (Fig. 3a). In addition, examples of the NB-UVB-reversed plaque-specific DEGs are shown in Fig. 3b. NB-UVB reversed a small number of plaque-specific DEGs in, both, the PE and CE skin. Some core plaque-specific DEGs may be affected either in PE or CE. skin.

#### Inflammatory regulator activity was suppressed and anti-inflammatory regulator activity was restored by NB-UVB, affecting their downstream genes

**Figure 4:**
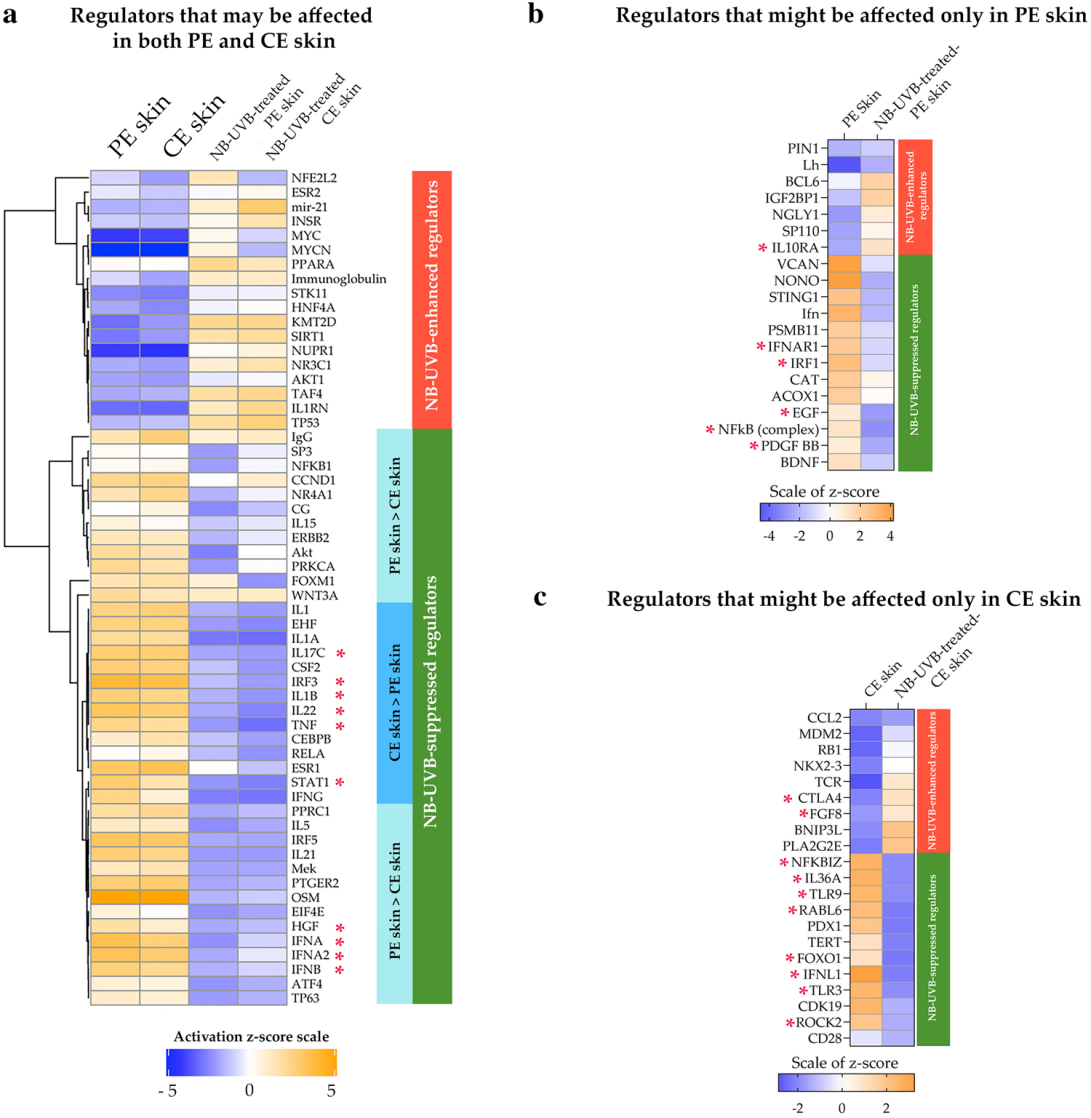
Effect of NB-UVB on potential upstream regulators. (**a**) Regulators that may be altered in both PE and CE skin. This cluster heatmap was created by “pheatmap” package in R software 1.0.12. (**b**–**c**) Regulators that might be altered either in PE skin or CE skin. Upstream regulators were analyzed by the upstream analysis function of QIAGEN’s IPA with a *p*-value of overlap < 0.05. The results are presented as a heatmap of the activation z-score. *indicates the potential key regulators. *CE skin* Center of lesional skin, *NB-UVB* Narrow band ultra violet B, *PE skin* Peripheral edge of lesional skin.

Upstream regulators control their respective downstream molecules. Thus, we analyzed alterations in the regulator’s activity after NB-UVB treatment. Potential regulators with their activity z score of untreated plaques were analyzed from bPE/UN DEGs and bCE/UN DEGs and of NB-UVB-treated plaques were analyzed from aPE/bPE DEGs and aCE/bCE DEGs. The results are represented as a heatmap of activation z score (Fig. 4a–c). NB-UVB suppressed the activation z-score of type I IFNs (IFN-α, IFN-α2, and IFN-β) more on the PE skin, while that of Type II IFN (IFN-γ) more on the CE skin. In addition, IFN α and β receptor subunit 1 (IFN-αR1), which is essential in Type I signaling^22^, was suppressed in the PE skin. Apart from the inflammation-related molecules, anti-inflammatory molecules, such as IL-10 receptor subunit α (IL-10Rα)^23^ and Cytotoxic T-lymphocyte antigen 4 (CTLA-4)^24,25^, were restored after NB-UVB treatment.

#### Mechanistic networks of NB-UVB were mediated through, both, plaque specific and non-plaque-specific DEGs, suppressing inflammation and abnormal growth

**Figure 5:**
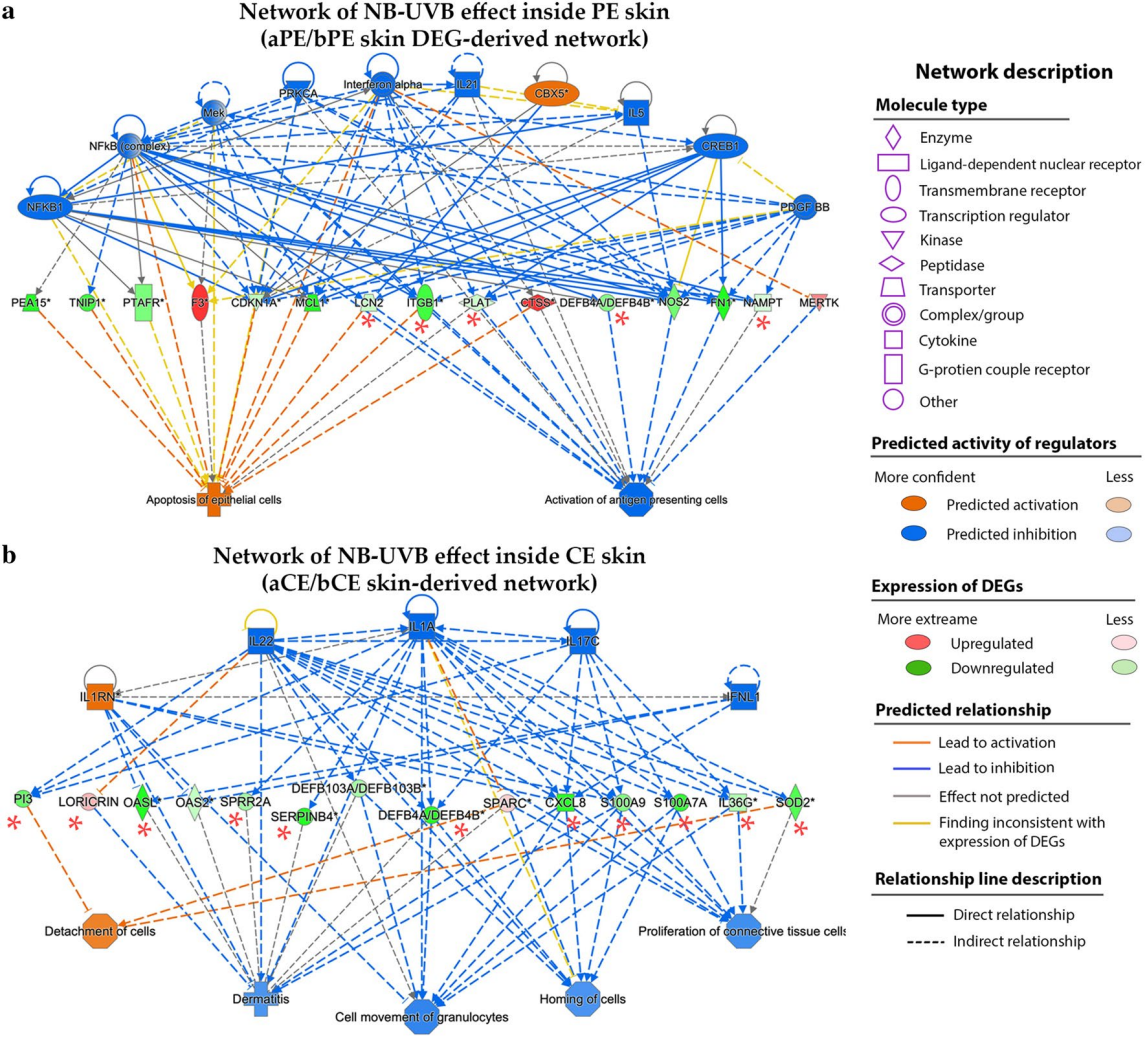
Potential networks for the molecular effect of NB-UVB. (**a**) Molecular network of NB-UVB effect in the PE skin. (**b**) Molecular network of NB-UVB effect in the CE skin. *indicates plaque-specific DEG. Regulator effect function of QIAGEN’s IPA was used to analyze the networks by overlapping the upstream regulators molecules to DEGs and their downstream functions. *aCE skin* After-treatment CE skin, *aPE skin* After-treatment PE skin, *bCE skin* Before-treatment CE skin, *bPE skin* Before-treatment PE skin, *DEGs* Differentially expressed genes, *NB-UVB* Narrow band ultra violet B.

Finally, we proposed the network of molecular effects after NB-UVB treatment in PE and CE skin by correlating the upstream regulator molecules to DEGs and their downstream functions (Fig. 5a–b). The mechanistic network of NB-UVB on the CE skin was primarily mediated through plaque-specific DEGs, in contrast to that on the PE skin.

## Discussion

**Figure 6:**
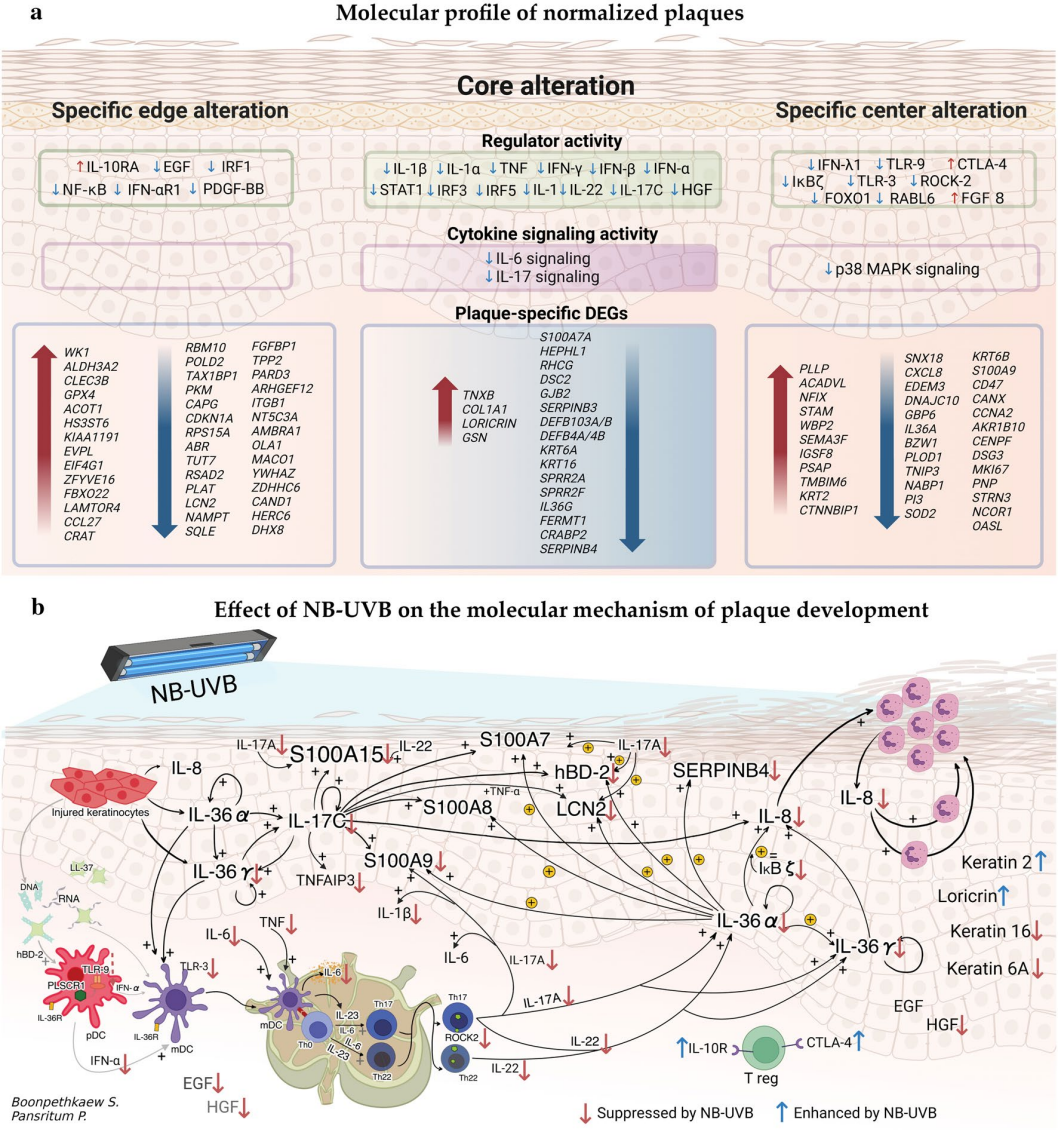
Molecular profile of normalized plaques and potential NB-UVB mechanism. (**a**) Molecular profile of normalized plaques based on RNA-seq analysis. (**b**) NB-UVB treatment suppressed the initial molecules, and molecules along the following cascade, resulting in the suppression of inflammation and abnormal proliferation. In addition, NB-UVB enhanced the normal differentiation/proliferation of keratinocytes and enhanced immunosuppressive function. Figure 6b was created by Adobe photoshop and BioRender.com. *CE skin* Center of lesional skin, *NB-UVB* Narrow band ultra violet B, *PE skin* Peripheral edge of lesional skin.

NB-UVB is the standard treatment for moderate to severe psoriasis. Its mechanism has continuously been elucidated. To our knowledge, this is the first report using RNA-seq technology with an advanced bioinformatic program to summarize the molecular profile in normalized plaques and to propose the mechanism of NB-UVB treatment by comparing the molecular changes in the PE and CE skin. The profile is shown in Fig. 6a and is discussed as follows.

Comparative analysis before and after treatment revealed that NB-UVB could alter more than 200 genes in, both, the PE and CE skin. However, approximately a quarter of these altered genes were plaque-specific DEGs. Among these, only 20 DEGs were affected by NB-UVB in both PE and CE skin (core alteration). The core NB-UVB downregulated DEGs included a few psoriasis signature genes, such as *IL36G*, *DEFB4A/B* (coding human β defensin 2), *S100A15*, *SERPINB4*, *KRT16*, *KRT6A,* and *DSC2*^20^. These downregulated genes suppressed chemotaxis, abnormal keratinization, and growth/proliferation of connective tissues in the plaques. In addition, some of these core NB-UVB-downregulated DEGs, such as *IL36G* and *DEFB4A/B*, were involved in IL-6 and IL-17 signaling (Fig. 3b), which are key cytokines in psoriasis pathogenesis. This might result in the attenuation of IL-6 and IL-17 signaling inside the treated plaques as well.

Proinflammatory IL-6 and TNF released by activated keratinocytes mature and activate mDCs to secrete IL-23^26–29^. Both IL-6 and IL-23 are cytokines in the microenvironment of Th17 differentiation^28,30^. In addition, Th22 needs IL-6 in the microenvironment for differentiation^28^. NB-UVB suppresses IL-6 mRNA with its protein secretion from peripheral blood monocytes of patients with psoriasis^31,32^ and could suppress IL-6 and TNF-α levels in suctioned blister fluid of treated plaques^33^. Thus, the suppression of activation z score for IL-6 signaling after NB-UVB treatment from PE to CE skin may have suppressed initial inflammation and the transitional step to maintenance phase.

IL-17A, also known as IL-17, is a key cytokine in the pathogenesis of psoriasis and Th17 is its primary source. Th17 is activated by mDC-derived IL-23 to release IL-17A. In addition to Th17, T cytotoxic (Tc)-17, γδ T cells, innate lymphoid cells, and NK T cells could also secrete IL-17A. IL-17A induces diverse proinflammatory cytokines (e.g., IL-1β, IL-6), antimicrobial peptides (AMPs) (e.g., DEFB4A, S100A9), and neutrophil chemokines (e.g., CXCL8 (IL-8)) from keratinocytes^27,28,34–36^. Our results revealed that *DEFB4A*, *S100A9*, and *CXCL8* were downregulated by NB-UVB treatment and the activity z-score of IL-1β (a regulator in both PE and CE skin) was decreased by NB-UVB treatment. In addition to IL-17A, IL-17C, another member of the IL-17 cytokine family, was also detected in psoriatic plaques and plays an important role in pathogenesis; NB-UVB treatment was found to decrease its activity z score. IL-17C is encoded by *IL17C,* which is a psoriasis signature gene^20^. Unlike IL-17A, IL-17C is majorly secreted by keratinocytes and acts as a regulator in, both, PE and CE skin^18,19^. It induces the expression of other psoriasis signature genes, such as *IL36G*, *S100A15*, *DEFB4A*, *S100A9*, *CXCL8*, *TNIP3* (coding TNF-α-induced-protein 3 or TNFAIP3), and *LCN2* (by synergy of IL-17C with TNF-α) in keratinocytes^18,20^. This study revealed that NB-UVB treatment suppressed these genes, particularly *IL36G*, *S100A15*, and *DEFB4A*, which were the core alteration.

In addition, various upstream regulators, such as TNF, IFN-α/β/γ, and IL-22, were altered after NB-UVB treatment in, both, PE and CE skin. However, several regulators were altered either in the PE skin (e.g., suppression of epidermal growth factor and Nuclear Factor Kappa B and enhancement of IL-10Rα) or CE skin (e.g., suppression of NF-Kappa-B Inhibitor Zeta and enhancement of CTLA-4).

Type I and II IFN signaling pathways are suppressed in psoriatic plaques after NB-UVB treatment^13^. Interestingly, NB-UVB treatment showed higher suppression of type I IFN (IFN-α) expression in the PE skin while increased suppression of type II IFN (IFN-γ) expression was observed in the CE skin. In addition to IL-6 and TNF secreted by activated keratinocytes, IFN-α secreted by plasmacytoid DCs could also activate mDCs, resulting in IL-23 production^27,29^. Thus, IFN-α also plays a role in initial inflammation and acts as an upstream cytokine along the IL-23/IL-17 axis in the pathogenesis^29^. Before IL-23/IL-17 axis had been established as the central mechanism, the IL-12/IFN-γ axis was primarily considered critical in psoriasis^29^. IFN-γ (type II IFN) could be secreted by Th1, T cytotoxic 1(Tc1), NK cells, and NK T cells, but not by keratinocytes, like IFN-α. Notably, Th17 is also capable of co-secreting IFN-γ with IL-17, particularly when stimulated with IL-12. IFN-γ has been also considered as upstream cytokine of the IL-23/IL-17 axis, wherein it stimulates mDCs to secrete IL-23^28,29,37^. Although IFN-γ plays a potential role in initial inflammation, blocking the IL-12/IFN-γ axis has limited beneficial effects^29^. However, IFN-γ mRNA expression is decreased in NB-UVB-normalized plaques^38^. In addition, our results suggested that NB-UVB tended to suppress the activity z-score of IFN-γ more in the CE skin than that in the PE skin. Accordingly, IFN- γ may be involved in the initial step latter than IFN-α.

Although it is difficult to identify the first molecule being altered in the normalized plaques (Fig. 6a), NB-UVB has the potential to suppress various molecules and signaling along the chronological mechanism of psoriatic plaque development^18,19^ (Fig. 6b). These suppress, both, inflammation-and growth-related downstream functions in the PE and CE skin; however, downstream functions related to inflammation are more likely to be suppressed in the PE skin than that in the CE skin, and those related to growth are more likely to be suppressed in the CE skin than that in the PE skin, which is in line with the concept of chronological plaque development^18,19^. With more than 75% staining of the proliferation marker protein Ki-67 of suprabasal/total epidermal, psoriasis is suggested over other psoriasiform dermatitis^39^. The study findings suggested that *MKI67* coding this marker was downregulated by NB-UVB treatment in the CE skin. In addition, NB-UVB treatment decreased IL-22 regulator activity more in the CE skin than that in the PE skin. IL-22 is secreted by Th17, and exclusively by Th22 and Tc22. It enhances keratinocyte migration, increases epidermal thickness, and inhibits keratinocyte differentiation. Like IL-17, IL-22 also enhances inflammation by inducing various cytokines, chemokines, and AMPs^29^. Other regulators such as Rho-Associated Protein Kinase 2, which is involved in the differentiation and proliferation of keratinocytes^40^, and Forkhead Box Protein O1, which is involved in the motility of keratinocytes^41^, also show decreased activity z score by NB-UVB treatment in the CE skin.

In psoriatic plaques, keratinocyte stem cells are activated and proliferate rapidly, and the terminal differentiation process is incomplete. Levels of late differentiation markers, such as loricrin and filaggrin, and terminal differentiation markers, such as keratin 2 are decreased, while the levels of keratin 6 and keratin 16, which are expressed in activated keratinocytes, are increased^37^. Here, NB-UVB could downregulate *KRT6A* (coding keratin 6A, an isoform of keratin 6) and *KRT16* and restore *LORICRIN,* both, in the PE and CE skin, and upregulated *KRT2* in the CE skin. This suggested that NB-UVB normalized psoriatic plaques by promoting normal differentiation to complete terminal differentiation, particularly in the CE skin.

Regarding immune cells, the relative abundance of activated CD4^+^ T cells significantly decreased in the PE skin after NB-UVB treatment. Although RNA-seq analysis showed that the relative abundance of T regs in psoriatic plaques was unchanged after NB-UVB treatment, its function inside the plaques might be restored, as indicated by the increased activity z-scores of regulatory IL-10Rα and CTLA-4, after NB-UVB treatment in the PE and CE skin, respectively. CTLA-4, a co inhibitory molecule expressed on T regs, binds to specific molecules on antigen presenting cells to transmit inhibitory signals, thereby decreasing their ability to stimulate effector T cells^24,25^. Similar to programmed cell death protein 1 (PD-1), CTLA-4 also acts as an immune checkpoint that is a negative regulator of T cell immune function^42^. In a western diet-induced obese mouse model, anti-PD-1 intensely aggravated psoriasiform ear thickness^43^. In addition, IL-10R, expressed on T regs, is important for their immunoregulatory functions. In mouse models, IL-10R-deficient T regs fail to suppress Th17 response and fail to produce IL-10^44^ as well as T reg-specific IL-10Rα deficiency leads to spontaneous hyper-Th17 function^23^. Thus, the increased activity z-score of both CTLA-4 and IL-10Rα reflect the enhancement function of T regs, attenuating the Th17 response after NB-UVB treatment.

A drawback of this study is the limited number of study samples for RNA-seq analysis. A larger sample size for RNA-seq may provide a more precise gene profile. Analysis using techniques such as polymerase chain reaction, immunohistochemistry, or western blot of the potential molecules in the proposed mechanism may be targeted. Furthermore, cross-ethnic analysis could help identify different molecular mechanisms of pathogenesis and possible treatments in the future^45–48^.

In conclusion, NB-UVB normalizes psoriatic plaques by suppressing the expression of psoriasis signature genes, such as *IL36G, DEFB4A/B, S100A15, SERPINB4, KRT16,* and *KRT6A* in, both, the PE and CE skin. In addition, IL-6 and IL-17A signaling activity and the expression of various upstream molecules such as IFNs, IL-17C, and IL-22 were suppressed. In summary, we proposed that NB-UVB can potentially suppress molecules along the initial inflammatory stage and later maintenance stage during psoriatic plaque development. This discovery helps explain why NB-UVB is so effective in clinical practice and may help in the development of new therapeutic approaches in which NB-UVB treatment is included for patients with psoriasis or other inflammatory skin diseases.

## Methods

### Patients

We recruited patients with psoriasis vulgaris (aged ≥ 18 years), from Thailand, with a PASI score ≥ 10 (moderate to severe). Patients who had been treated with topical agents (steroids, vitamin D analogues) within 2 weeks or had undergone systemic medication (cyclosporin, methotrexate, biologics) and phototherapy within 4 weeks of the date of tissue sampling were excluded. A total of three patients participated in this study. The patients were informed of all study protocols, and they provided written informed consent. A graphic summary of the method is provided in supplementary Fig. S3a. This study was conducted according the Declaration of Helsinki guidelines and was approved by the Human Ethics Committee of Thammasat University (No. MTU-EC-OO-6-188/65).

### Treatment

The patients were treated with NB-UVB 2–3 times for 12 weeks with a starting dose 200 mJ/cm^2^. This dose was gradually increased 10–20% from the initial dose until the minimal erythema dose was met. Side effects of NB-UVB treatment included erythema, tenderness, and burning sensation; these were monitored as shown in supplementary Fig. S3b. All patients exhibited more than 75% reduction in the PASI score at 12 weeks (PASI 75). PASI scores before and after treatment are listed in supplementary Table S2.

### Tissue sampling

Lidocaine (2%) was used for pain control. Full thickness tissue samples were collected using a 6-mm punch biopsy equipment. UN skin, 10 cm away from the edge, was biopsied before treatment. The PE and CE skin were biopsied from active lesion before treatment (first biopsy) and 7 days after treatment (second biopsy). To certify that the after-treatment biopsies were from the same area of preexisting lesions, the shape of the selected lesions was drawn on clear plastic sheaths, which were used as a reference for the second biopsy (Fig. S3C). All biopsies were preserved in RNAlater (ThermoFisher, AMBION, USA) at − 80 °C until further analysis.

### High-throughput next-generation sequencing

Based on previous RNA-seq profiling studies in human psoriasis skin, three samples were deemed to be adequate for analysis^17,49^. Therefore, the UN skin, bPE skin, aPE skin, bCE skin, and after aCE skin samples from three patients were used for RNA-seq. Total RNA was extracted from each sample (bPE, aPE, bCE, aCE, UN skin, n = 2) using the TRIzol Reagent (Ambion, Austin, TX, USA). Next-generation sequencing (NGS) was performed using Macrogen (Seoul, Korea). Briefly, the structural integrity and purity of RNA were analyzed using an Agilent 2100 Bioanalyzer (RNA integrity number > 7). Sequence libraries were constructed using the SMARTer Universal Low Input RNA Kit and TruSeq RNA Sample Prep Kit v2 (pair-end). NGS was performed using the NovaSeq 6000 S4 Reagent Kit on the NovaSeq 6000 System (Illumina Inc., San Diego, CA, USA) with 100-bp paired-end reads. The raw data generated in this study have been deposited in the NCBI Gene Expression Omnibus (GEO; http://www.ncbi.nlm.nih.gov/geo) and are accessible through the GEO Series accession number GSE183732 and GSE186117 for UN skin, PE skin, CE skin transcripts before treatment and GSE179632 and GSE179731 for PE skin and CE skin transcript after treatment.

### Sequencing data analysis

Basic data analysis was conducted using Macrogen. Briefly, overall read qualities, total bases, total reads, % guanine-cytosine content, and basic statistics were calculated. Trimmed reads were mapped to the reference genome using HISAT2, a splice-aware aligner. The transcripts were assembled using StringTie with aligned reads. Expression profiles were repeated as read counts and normalized based on the transcript length and depth of coverage. The fragments per kilobase of transcript per million mapped reads value was used for normalization. Furthermore, we used the read counts to statistically analyze the expression profile with edgeR Bioconductor statistical library version 3.13^50,51^ on R Studio^52^.

### DEG analysis

The DEGs were determined according to *p*-value < 0.01 and FC ratio (|log2FC|) ≥ 1.5. QIAGEN’s IPA software (QIAGEN Redwood City, www.qiagen.com/ingenuity) was used as the “core analysis” of DEGs in aspects of disease and biofunction, canonical pathways, and upstream regulators. The Metascape online tool (https://metascape.org/gp/index.html) was additionally used to analyze functional enrichment.

### Graphic presentation

GraphPad prism 9.4.1 and Adobe photoshop 2021 were used to created graphical presentations. Figure 6 was created with BioRender.com and Adobe photoshop.

### Supplementary Information

Supplementary Information.

## Data Availability

The raw data generated in this study have been deposited in the NCBI Gene Expression Omnibus (GEO; http://www.ncbi.nlm.nih.gov/geo) and are accessible through the GEO Series accession number GSE183732, GSE186117, GSE179632 and GSE179731.
