# Author Correction: Elucidating the NB-UVB mechanism by comparing transcriptome alteration on the edge and center of psoriatic plaques

**DOI:** 10.1038/s41598-023-42859-8

**Published:** 2023-09-22

**Authors:** Suphagan Boonpethkaew, Jitlada Meephansan, Sasin Charoensuksira, Onjira Jumlongpim, Pattarin Tangtanatakul, Jongkonnee Wongpiyabovorn, Mayumi Komine, Akimichi Morita

**Affiliations:** 1 Division of Dermatology, Chulabhorn International College of Medicine, Thammasat University, Rangsit Campus, Klong Luang, Pathum Thani 12120 Thailand; 2 Department of Transfusion Medicine and Clinical Microbiology, Faculty of Allied Health Sciences, Chulalongkorn University, Bangkok, 10330 Thailand; 3 Department of Microbiology, Faculty of Medicine, Center of Excellence in Immunology and Immune-Mediated Disease, Chulalongkorn University, Bangkok, 10330 Thailand; 4 Department of Dermatology, Jichi Medical University, 3311-1 Yakushiji, Shimotsuke, Tochigi 329-0498 Japan; 5 Department of Geriatric and Environmental Dermatology, Nagoya City University Graduate School of Medical Sciences, Nagoya, 467-8601 Japan

Correction to: *Scientific Reports* 10.1038/s41598-023-31610-y, published online 16 March 2023

The original version of this Article contained an error in the name of author Akimichi Morita, which was incorrectly given as Morita Akimichi.

The original Article has been corrected.

