# Supplementary material for: Elucidating the NB-UVB mechanism by comparing transcriptome alteration on the edge and center of psoriatic plaques
Source: Sci Rep. 2023 Mar 16;13:4384. doi: 10.1038/s41598-023-31610-y (PMC10020439; doi:10.1038/s41598-023-31610-y)
Supplement: Supplementary file 1 — Supplementary Information. [file 41598_2023_31610_MOESM1_ESM.docx]

**Supplementary Information**

**Supplementary Fig. S1** NB-UVB-altered plaque-specific DEGs of, both, PE and CE skin.

**(a)** Heatmap showing the 62 and 65 plaque-specific DEGs of PE skin and **(b)** CE skin, respectively, which were altered after NB-UVB treatment. aCE skin, after-treatment CE skin; aPE skin, after-treatment PE skin; bCE skin, before-treatment CE skin; bPE skin, before-treatment PE skin; DEGs, differentially expressed genes; FC, fold change; NB-UVB, narrow band ultra violet B; UN skin, uninvolved skin.


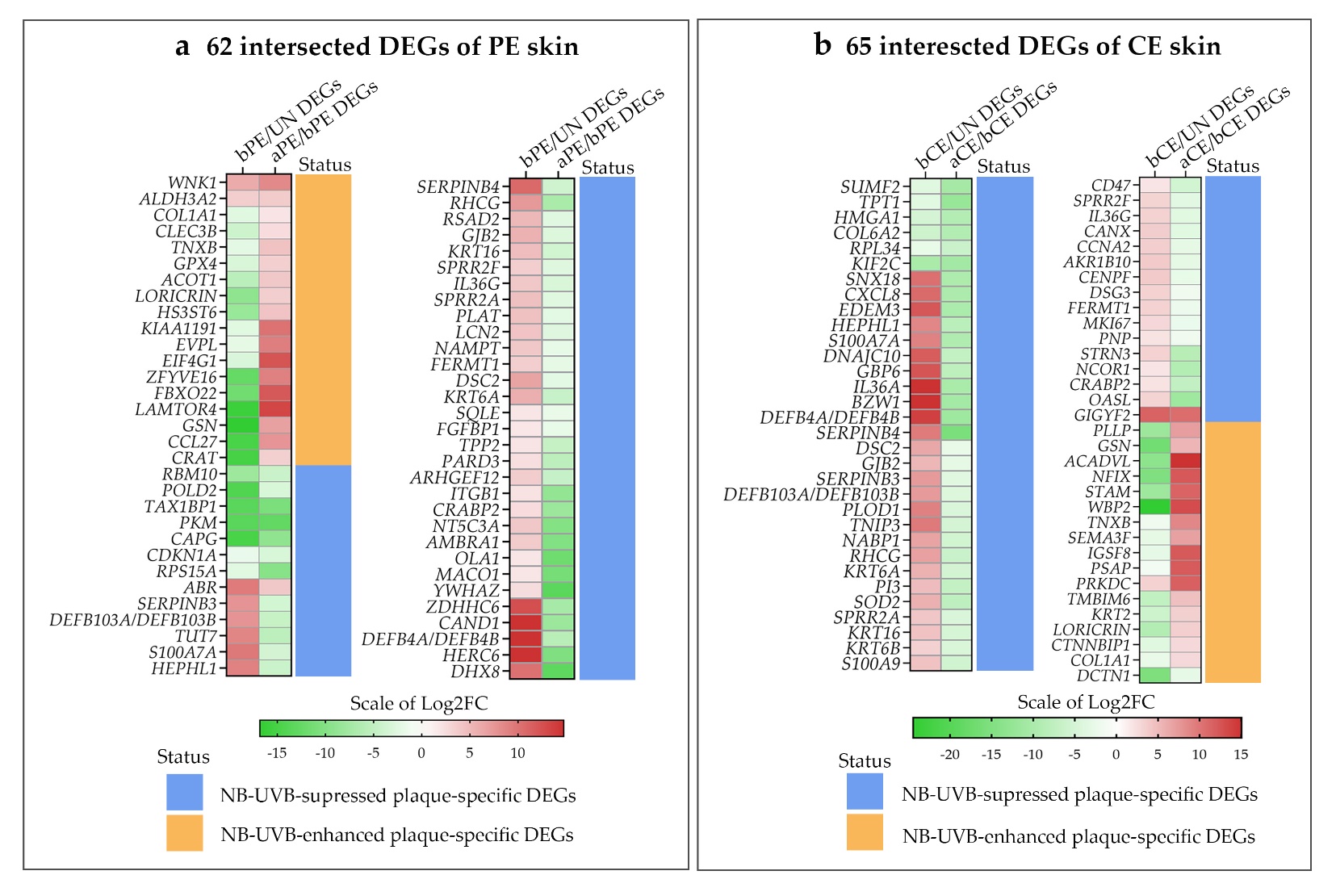


**Supplementary Fig. S2** Biofunction and canonical pathway analysis.

Untreated group (bPE/UN-, bCE/UN-, and bPE/bCE skin-derived DEGs) and NB-UVB-treated group (aPE/bPE-, aCE/bCE, and aPE/aCE skin-derived DEGs) were analyzed for their functional and canonical pathway enrichments by QIAGEN’s Ingenuity Pathway Analysis. **(a)** DEG-enriched functions and **(b)** DEG-enriched canonical pathways of cytokines are shown with a heatmap of activation z-score. Orange, white, and blue color represent activation, neutral, and inhibition. A dot is designated for │z score│ < 1. aCE skin, after-treatment CE skin; aPE skin, after-treatment PE skin; bCE skin, before-treatment CE skin; bPE skin, before-treatment PE skin; DEGs, differentially expressed genes; NB-UVB, narrow band ultra violet B; UN skin, uninvolved skin.


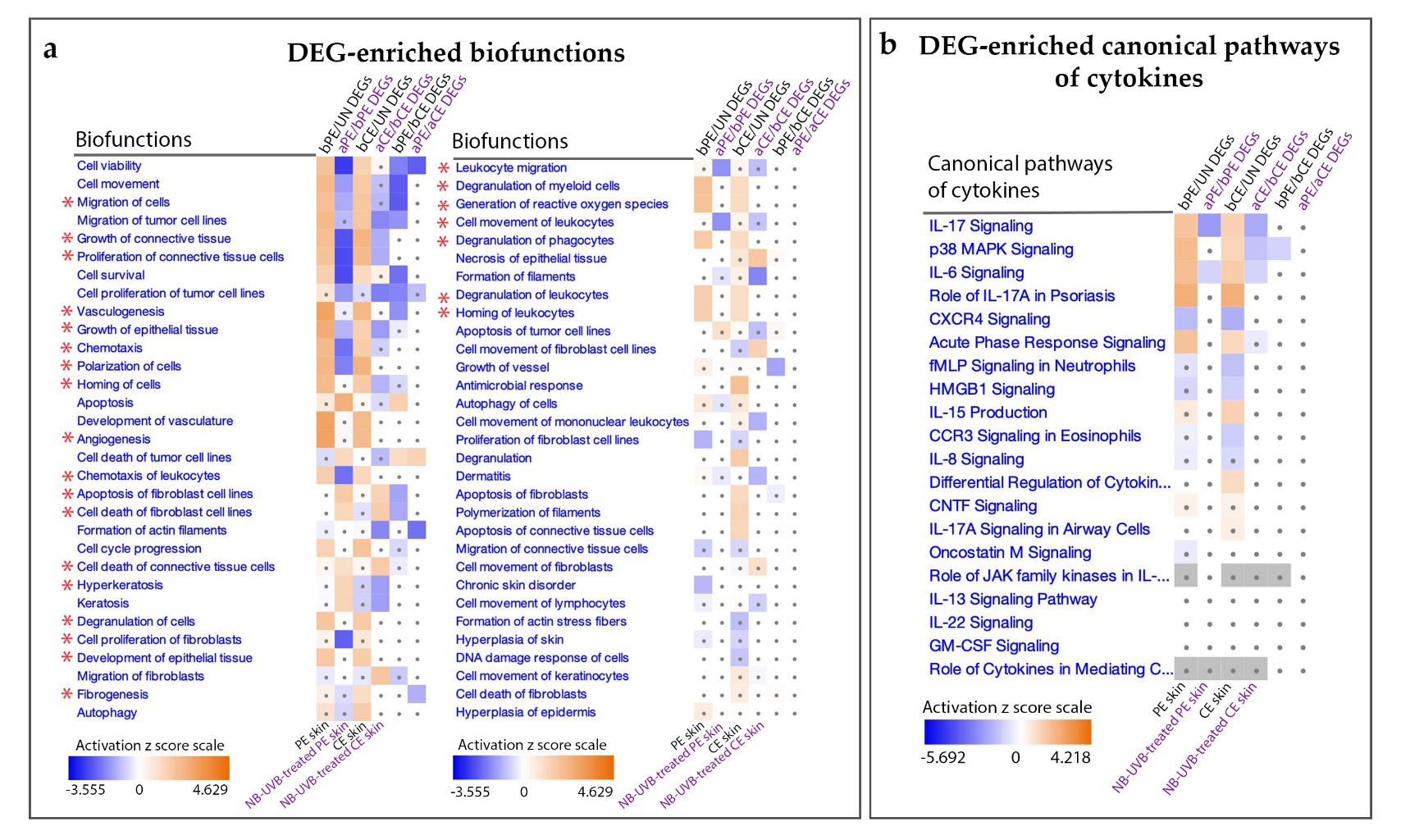


**Supplementary Fig. S3** Method and research design

**(a)** Flowchart for the study design. **(b)** A time-line tracing the beginning to end of intervention (NB-UVB treatment) and tissue sampling. **(c)** A diagram showing the biopsy landmark. Size and site of biopsy were marked on transparent paper used as the reference for the second biopsy after NB-UVB treatment. aCE skin, after-treatment CE skin; aPE skin, after-treatment PE skin; bCE skin, before-treatment CE skin; bPE skin, before-treatment PE skin; NB-UVB, narrow band ultra violet B; PASI, psoriasis area severity index; UN skin, uninvolved skin. This figure was created with Adobe photoshop and BioRender.com


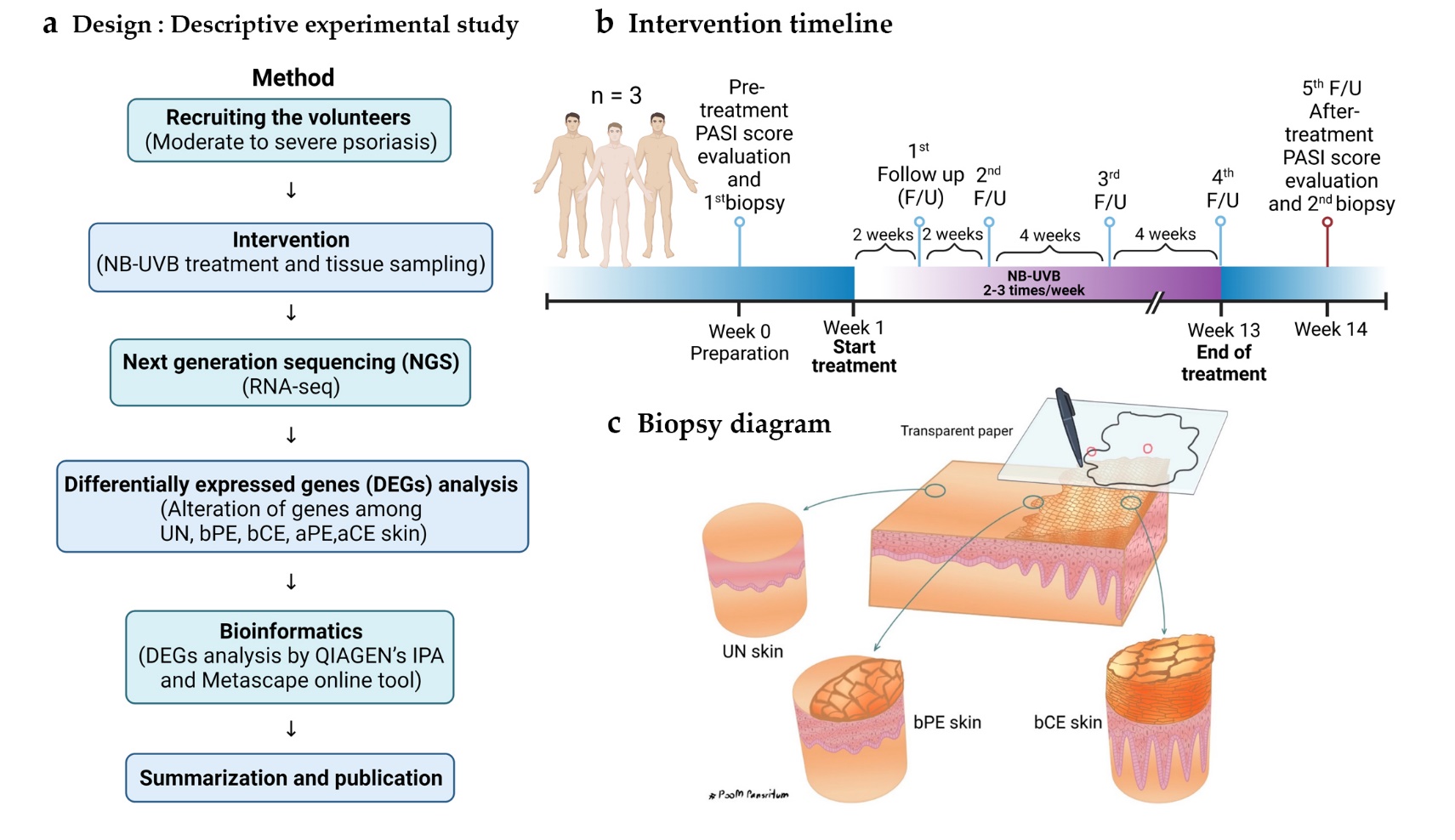


| **Category**  **Supplementary Table S1.** The list of all NB-UVB-altered plaque-specific DEGs in each function and pathway | **Term** | **Description** | **Log *p*-value** | **NB-UVB-altered plaque-specific DEGs** |
| --- | --- | --- | --- | --- |
| **NB-UVB-suppressed functions and pathways in PE skin** | | | | |
| Reactome Gene Sets | R-HSA-6803157 | Antimicrobial peptides | -8.47 | *DEFB4A,LCN2,DEFB103B,S100A7A,DEFB103A,DEFB4B,KRT16,KRT6A,SPRR2A,GJB2,HERC6,IL36G,ITGB1,SERPINB3,NAMPT,MACO1* |
| Reactome Gene Sets | R-HSA-6809371 | Formation of the cornified envelope | -6.04 | *DSC2,KRT6A,KRT16,SPRR2A,SPRR2F,CRABP2,RHCG* |
| GO Biological Processes | GO:0090303 | positive regulation of wound healing | -4.06 | *ITGB1,PLAT,FERMT1,KRT16,PARD3,SERPINB3,FGFBP1* |
| Reactome Gene Sets | R-HSA-9013106 | RHOC GTPase cycle | -3.85 | *ABR,ARHGEF12,MACO1,ITGB1,PARD3,YWHAZ,CDKN1A* |
| Reactome Gene Sets | R-HSA-1280215 | Cytokine Signaling in Immune system | -3.42 | *CDKN1A,ITGB1,LCN2,YWHAZ,IL36G,RSAD2,KRT6A,PLAT,OLA1* |
| Canonical Pathways | M5885 | NABA MATRISOME ASSOCIATED | -3.30 | *PLAT,SERPINB3,SERPINB4,FGFBP1,IL36G,S100A7A* |
| WikiPathways | WP2877 | Vitamin D receptor pathway | -2.69 | *CDKN1A,DEFB4A,KRT16* |
| GO Biological Processes | GO:0031334 | positive regulation of protein-containing complex assembly | -2.65 | *FERMT1,AMBRA1,CAND1* |
| GO Biological Processes | GO:0006605 | protein targeting | -2.32 | *YWHAZ,PARD3,ZDHHC6* |
| **NB-UVB-enhanced functions and pathways in PE skin** | | | | |
| GO Biological Processes | GO:0032787 | monocarboxylic acid metabolic process | -5.84 | *ALDH3A2,CRAT,GPX4,PKM,TNXB,ACOT1* |
| GO Biological Processes | GO:0031669 | cellular response to nutrient levels | -3.26 | *COL1A1,EIF4G1,FBXO22* |
| GO Biological Processes | GO:0097435 | supramolecular fiber organization | -3.14 | *CAPG,COL1A1,GSN,TNXB,CCL27,LAMTOR4,EIF4G1* |
| GO Biological Processes | GO:0043588 | skin development | -3.01 | *COL1A1,EVPL,LORICRIN,ALDH3A2* |
| KEGG Pathway | hsa05165 | Human papillomavirus infection | -2.71 | *COL1A1,PKM,TNXB,LAMTOR4* |
| GO Biological Processes | GO:0045862 | positive regulation of proteolysis | -2.57 | *GSN,CLEC3B,FBXO22* |
| GO Biological Processes | GO:0030335 | positive regulation of cell migration | -2.07 | *COL1A1,CCL27,WNK1* |
| **NB-UVB-suppressed functions and pathways in CE skin** | | | | |
| GO Biological Processes | GO:0009617 | response to bacterium | -11.05 | *DEFB4A,GJB2,CXCL8,KRT6A,PI3,S100A9,SOD2,SPRR2A,IL36A,DEFB103B,IL36G,TNIP3,GBP6,DEFB103A,KRT16* |
| Reactome Gene Sets | R-HSA-6809371 | Formation of the cornified envelope | -10.36 | *DSC2,DSG3,KRT6A,KRT6B,KRT16,PI3,SPRR2A,SPRR2F,CRABP2,PLOD1,RHCG* |
| Reactome Gene Sets | R-HSA-6803157 | Antimicrobial peptides | -9.66 | *DEFB4A,PI3,S100A9,DEFB103B,S100A7A,DEFB103A,DEFB4B,KRT6A,SPRR2A,GBP6,CXCL8,KRT16,SERPINB3* |
| GO Biological Processes | GO:0032496 | response to lipopolysaccharide | -6.04 | *GJB2,CXCL8,S100A9,SOD2,IL36A,IL36G,TNIP3,PI3,PLOD1,SERPINB3,SERPINB4,S100A7A,CANX,OASL,GBP6,CCNA2,KRT16,CD47* |
| GO Biological Processes | GO:0032355 | response to estradiol | -4.28 | *CCNA2,GJB2,SPRR2A,STRN3* |
| GO Biological Processes | GO:0007568 | aging | -3.79 | *CANX,GJB2,KRT16,SOD2* |
| GO Biological Processes | GO:1903047 | mitotic cell cycle process | -3.70 | *CCNA2,CENPF,KIF2C,GIGYF2,NABP1,SNX18,MKI67* |
| GO Biological Processes | GO:0030433 | ubiquitin-dependent ERAD pathway | -3.48 | *CANX,DNAJC10,EDEM3,CXCL8* |
| Canonical Pathways | M3468 | NABA ECM REGULATORS | -3.19 | *PI3,PLOD1,SERPINB3,SERPINB4,CXCL8,S100A9* |
| GO Biological Processes | GO:1990748 | cellular detoxification | -3.15 | *S100A9,SOD2,AKR1B10,TPT1,RHCG,HEPHL1* |
| Reactome Gene Sets | R-HSA-2262752 | Cellular responses to stress | -2.74 | *CCNA2,HMGA1,CXCL8,RPL34,SOD2,NCOR1* |
| GO Biological Processes | GO:1902532 | negative regulation of intracellular signal transduction | -2.68 | *SERPINB3,SOD2,TPT1,NCOR1,TNIP3* |
| GO Biological Processes | GO:0030335 | positive regulation of cell migration | -2.64 | *CD47,CXCL8,SERPINB3,SOD2,FERMT1,S100A9,OASL,PNP* |
| GO Biological Processes | GO:0007626 | locomotory behavior | -2.45 | *SOD2,NCOR1,GIGYF2,CXCL8,PNP* |
| **NB-UVB-enhanced functions and pathways in CE skin** | | | | |
| GO Biological Processes | GO:0097435 | supramolecular fiber organization | -5.00 | *COL1A1,DCTN1,GSN,KRT2,TNXB* |
| GO Biological Processes | GO:0030855 | epithelial cell differentiation | -3.59 | *ACADVL,KRT2,LORICRIN,PSAP,COL1A1* |
| GO Biological Processes | GO:0070925 | organelle assembly | -3.09 | *DCTN1,GSN,PRKDC,STAM,TMBIM6* |
| GO Biological Processes | GO:0003002 | regionalization | -3.09 | *PRKDC,SEMA3F,CTNNBIP1,DCTN1,GSN* |
| Reactome Gene Sets | R-HSA-5653656 | Vesicle-mediated transport | -2.23 | *COL1A1,DCTN1,STAM,WBP2,PRKDC* |
| *Blue* represents NB-UVB-suppressed plaque-specific DEGs and *orange* represents NB-UVB-enhanced plaque-specific DEGs.  DEGs, differentially expressed genes; NB-UVB, narrow band ultra violet B. | | | | |

|  | |  |  |  |  |  |  |  |  |  |  |
| --- | --- | --- | --- | --- | --- | --- | --- | --- | --- | --- | --- |
| **Supplementary Table S2** Patients characteristics | | | | | | | | | | | |
| **Patient** | **Age** | | **Sex** | **BMI**  **(kg/m^2^)** | **Skin lesion** | **Scalp lesion** | **Nail lesion** | **Before NB-VUB PASI** | **After NB-UVB PASI** | **PASI reduction** | **Side effect of NB-UVB** |
| 1 | 57 | | Male | 24.4 | ✓ | ✓ | ✓ | 12.5 | 2 | 84% | Dry skin and erythema |
| 2 | 23 | | Male | 20.3 | ✓ | ✓ | ✓ | 12.5 | 1 | 92% | Dry skin and erythema |
| 3 | 39 | | Male | 20.5 | 🗶 | ✓ | ✓ | 35.5 | 5 | 86% | Dry skin and erythema |
